# Patterns of Reproductive Isolation in Toads

**DOI:** 10.1371/journal.pone.0003900

**Published:** 2008-12-09

**Authors:** John H. Malone, Brian E. Fontenot

**Affiliations:** Department of Biology, The University of Texas at Arlington, Arlington, Texas, United States of America; American Museum of Natural History, United States of America

## Abstract

Understanding the general features of speciation is an important goal in evolutionary biology, and despite significant progress, several unresolved questions remain. We analyzed an extensive comparative dataset consisting of more than 1900 crosses between 92 species of toads to infer patterns of reproductive isolation. This unique dataset provides an opportunity to examine the strength of reproductive isolation, the development and sex ratios of hybrid offspring, patterns of fertility and infertility, and polyploidization in hybrids all in the context of genetic divergence between parental species. We found that the strength of intrinsic postzygotic isolation increases with genetic divergence, but relatively high levels of divergence are necessary before reproductive isolation is complete in toads. Fertilization rates were not correlated to genetic divergence, but hatching success, the number of larvae produced, and the percentage of tadpoles reaching metamorphosis were all inversely related with genetic divergence. Hybrids between species with lower levels of divergence developed to metamorphosis, while hybrids with higher levels of divergence stopped developing in gastrula and larval stages. Sex ratios of hybrid offspring were biased towards males in 70% of crosses and biased towards females in 30% of crosses. Hybrid females from crosses between closely related species were completely fertile, while approximately half (53%) of hybrid males were sterile, with sterility predicted by genetic divergence. The degree of abnormal ploidy in hybrids was positively related to genetic divergence between parental species, but surprisingly, polyploidization had no effect on patterns of asymmetrical inviability. We discuss explanations for these patterns, including the role of Haldane's rule in toads and anurans in general, and suggest mechanisms generating patterns of reproductive isolation in anurans.

## Introduction

Reproductive isolation is a defining characteristic of a biological species, and it is integral to creating and maintaining species boundaries [1]. Breakdown in reproductive isolation can lead to gene flow between species, resulting in sterility or inviability of hybrid offspring, genetic assimilation of the rarer species, introduction of novel genetic variation, reinforcement of species boundaries, increased or decreased fitness of hybrids in natural environments, ploidy changes, and rapid speciation [2]–[4].

A major goal of speciation research is to understand the factors that promote reproductive isolation between species. One powerful and widely used approach is to use comparative analyses of interspecific hybridization to identify patterns of reproductive isolation [1]. Comparative analyses of reproductive isolation have been conducted on a variety of organisms including fungi [5], orchids [6], angiosperms [7], worms [8], flies [9]–[11], mosquitoes [12], [13], butterflies and moths [14], frogs [15], birds [16]–[18], fish [19]–[22], and mammals [23], [24]. These analyses have used measures of genetic distance as a surrogate for divergence time between species to examine the relationship between genetic divergence and reproductive isolation. The broad patterns produced from these studies can be summarized as follows: 1) hybrid sterility evolves faster than hybrid inviability, but both measures of reproductive isolation evolve gradually; 2) the time frame in which pre- or postzygotic isolation evolves between species is organism-specific; and 3) Haldane's rule is obeyed regardless of sex determination system (ZW or XY), and is an important first step toward the reproductive isolation of biological species [1], [25]–[27], [but see 28]. Furthermore, these comparative analyses have also been used to demonstrate that ecological divergence and reproductive isolation are strongly associated, suggesting that ecological adaptation plays an important role in reproductive isolation [29].

True toads of the genus *Bufo* (as formerly recognized, but recently split into several genera; see [30]) have never been used to examine patterns of reproductive isolation despite a wealth of knowledge regarding interspecific hybridization in this group [44]. Extant toads are diverse with more than 280 species distributed on all continents except Australia and Antarctica. Divergence estimates place the origin of the genus *Bufo* and the family Bufonidae in the upper Cretaceous between 78–99 million years ago, with a likely geographical origin in South America [31], [32]. While the origins of the group are relatively ancient, all major lineages of extant bufonids are believed to have dispersed from South America during the Eocene approximately 34–56 mya [31]–[32].

The life history characteristics of toads make them suitable organisms for reproductive isolation research. Toads are abundant and conspicuous in most habitats, easy to rear in lab environments, and interspecific hybridization is facile due to their external fertilization reproductive mode. Consequently, toads formerly served as models of vertebrate speciation due to the extensive studies of W. F. Blair [33]–[40].

From the 1950s to 1970s, Blair and colleagues performed more than 1,900 crosses between 92 species from all major clades of the family Bufonidae, and quantified several measures of postzygotic reproductive isolation. These efforts represent one of the most extensive hybridization datasets for any vertebrate, in terms of both the sheer number of crosses and the number of species used to make hybrids. Recent advances in bufonid systematics and phylogenetics [30], [32], [41]–[43] have produced a molecular dataset, which can be used to infer interspecific genetic divergence. Estimates of phylogeny and accompanying molecular data, combined with Blair's reproductive isolation work, provide the opportunity to perform comparative analyses of reproductive isolation in the family Bufonidae. Such analyses provide an opportunity to evaluate emergent patterns of reproductive isolation for the characteristics of speciation. Additionally, certain unique features of Blair's data also permit an exploration of previously unaddressed questions regarding the relationship between genetic divergence and development, biased sex ratios, fertility/infertility, and polyploidization in hybrid offspring.

For example, Blair and colleagues documented the stage of growth at which hybrid offspring ceased to develop. We used these data to explore the relationship between development and genetic divergence between parental species, and this fills an important gap in reproductive isolation research. Additionally, many hybrid individuals were preserved as museum specimens, and we examined the gonads to calculate sex ratios of hybrid offspring in an analysis of hybrid inviability. An analysis of sex ratios is of interest because the sex ratios of hybrid offspring are often biased in interspecific crosses. This pattern is manifested as Haldane's rule, one of the few rules of speciation. Haldane's rule states that if one sex is inviable or sterile in interspecific crosses, that sex is the heterogametic sex [25]. Blair's data provide an opportunity to evaluate the operation of Haldane's rule in an additional group of organisms.

Finally, and perhaps most interesting, are the extensive efforts by Blair and colleagues to document changes in chromosome content resulting from hybridization [44]. The relationship between ploidy changes during hybridization and genetic divergence has not previously been examined in animals, but could be germane to patterns of sterility and inviability. Most toads possess a diploid chromosome number of 22 with the exception of several species of African toads, such as *Bufo regularis*, which possess a diploid chromosome number of 20. These differences do not prevent 20 chromosome toads from producing viable and, in some cases, fertile hybrid offspring when crossed with 22 chromosome toads. Interestingly, members of the *Bufo viridis* complex consist of natural breeding populations of diploid, triploid, and tetraploid individuals [44], [45]. Bogart [44] described karyotype analyses on tadpoles produced from interspecific crosses, and we used these data to analyze the relationship between ploidy, genetic divergence, inviability, and sterility.

We used Blair's extensive dataset, one of the largest for any vertebrate organism, to test features of reproductive isolation and resolve questions that still remain about the broad features of speciation. We ask the following questions: 1) How does postzygotic reproductive isolation change with genetic divergence among toads?; 2) What is the relationship between development and genetic divergence in hybrids?; 3) What are the patterns of inviability and sterility when crossing different species of *Bufo*?; and 4) What is the relationship between genetic divergence and polyploidization, and can abnormal ploidy explain patterns of infertility?

## Results

### Patterns of Reproductive Isolation

We compiled the data found in Blair (1972) [40] which consisted of 1,934 crosses between 92 species of *Bufo*; one of the largest datasets for any vertebrate organism. Crosses were generally made by *in vitro* fertilization using squashes of testis from one species to fertilize the eggs of another species in a petri dish. Various measures of reproductive isolation were quantified based on these *in vitro* fertilizations including; 1) the percentage of fertilized eggs; 2) percentage of hatching embryos; 3) number of tadpoles produced; 4) percentage metamorphosed; 5) fertility in backcross analysis; and 6) the stage at which eggs ceased to develop. From these data, we developed an index of reproductive isolation to explore the relationship between reproductive isolation and genetic distance (Table 1 and see Methods). This index ranges from 0 (no reproductive isolation) to 1 (complete reproductive isolation), and allowed us to examine the relationship between genetic divergence and reproductive isolation [9], [10], [14], [15].

**Table 1:** Index of postzygotic reproductive isolation for reciprocal cross data (IPO). V/F, viable and/or fertile; I, inviable or sterile.

Sex	A		B		C		D		E		F	
	M	F	M	F	M	F	M	F	M	F	M	F
Cross 1	V/F	V/F	V/F	V/F	V/F	I	I	I	V/F	I	I	I
Cross 2	V/F	V/F	V/F	1	V/F	I	V/F	V/F	I	I	I	I
Code	0		1		2		3		4		5	
IPO	0.00		0.20		0.40		0.60		0.80		1.00	

(A) Both sexes viable or fertile in both reciprocal crosses; (B) one sex sterile or inviable in one cross only; (C) one sex inviable or sterile in both directions of cross; (D) both sexes inviable or sterile in only one direction of cross; (E) one sex viable or fertile in both directions of cross; (F) both sexes inviable or sterile in both directions of cross; M = males; F = females.

To measure genetic divergence between species, we downloaded sequence data for the mitochondrial DNA fragment 12S–16S from Genbank for 69 of the 92 species (Appendix S1; [30], [42], [43]), and calculated genetic distance for all pairwise comparisons. Genetic distances (uncorrected p) had a wide range, from 0.002–0.152 (mean = 0.083±0.001 SE) indicating that divergence between toad populations worldwide is substantial.

To ensure that our observations of reproductive isolation were phylogenetically and statistically independent, we employed Coyne and Orr's [9], [10] modification of Felsenstein's independent contrasts method (see Methods). First, we generated a phylogenetic tree using maximum parsimony methods to examine the phylogenetic relationships among toad species and then averaged the genetic distances and other measures of postzygotic isolation for species pairs that were not independent to produce a single, independent comparison.

**Figure 1:**
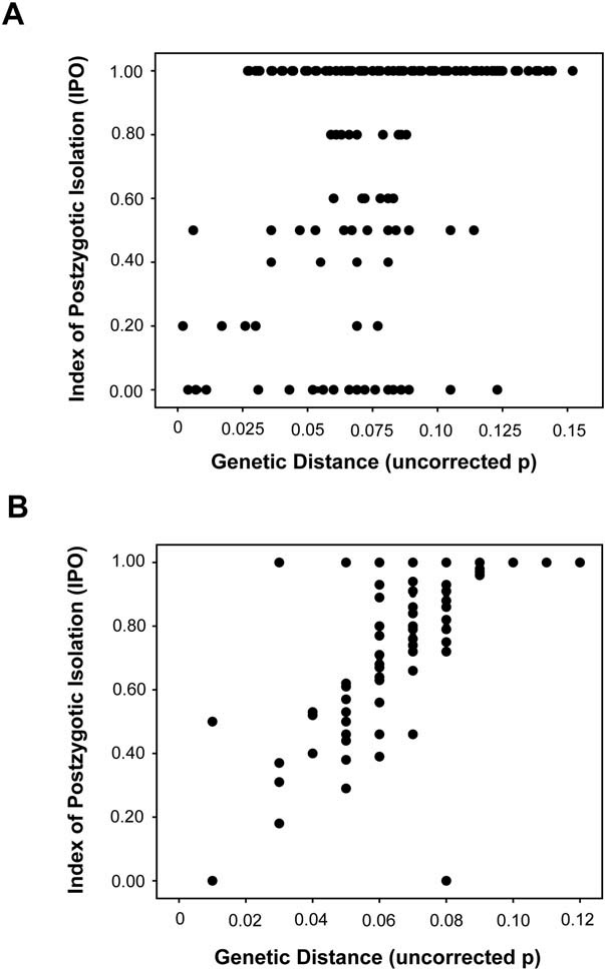
Scatterplot of postzygotic isolation indices and genetic distance (uncorrected p) of (A) the entire dataset (*r_s_* = 0.406, *N* = 680, *P*<0.001), and (B) corrected for phylogenetic independence (*r_s_* = 0.538, *N* = 101, *P*<0.001). Both show that postzygotic reproductive isolation increases with increasing genetic distance.

Genetic distance showed a significant positive correlation with reproductive isolation (for uncorrected data, Spearman rank correlation: *r_s_* = 0.406, *N* = 680, *P*<0.001; Fig. 1A; for corrected data, Spearman rank correlation: *r_s_* = 0.538, *N* = 101, *P*<0.001; Fig. 1B). Overall, the average genetic distance among species pairs was 0.067±0.002 and average postzygotic isolation index was 0.77 for the phylogenetically corrected dataset (*N* = 101). These results suggest that postzygotic isolation increases with genetic distance between diverging species in the family Bufonidae.

**Figure 2:**
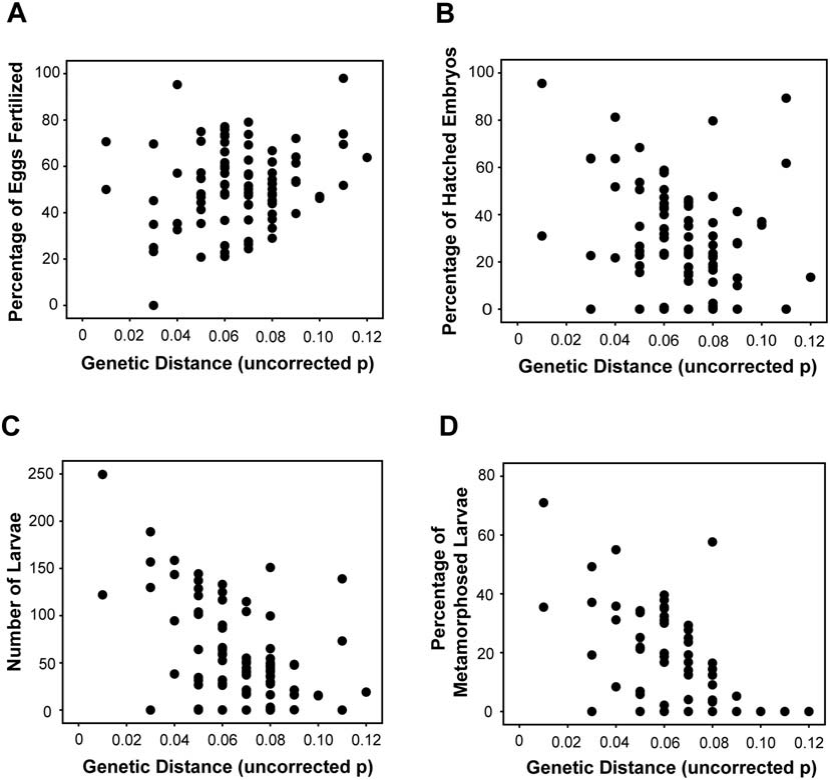
Scatterplot of (A) percentage of eggs fertilized (*r_s_* = 0.078, *N* = 101, *P*<0.437), (B) percentage of embryos hatched (*r_s_* = −0.246, *N* = 101, *P*<0.013), (C) the number of larvae (*r_s_* = −0.378, *N* = 101, *P*<0.001), and (D) the percentage of larvae that metamorphosed into froglets (*r_s_* = −0.499, *N* = 101, *P*<0.001) compared to genetic distance between species in interspecific hybrid crosses for the phylogenetically corrected dataset.

We also analyzed the relationship between various characteristics of postzygotic reproductive isolation and genetic divergence. The percentage of eggs that were fertilized in hybrid crosses was not related to genetic distance (*r_s_* = 0.078, *N* = 101, *P*<0.437; Fig. 2A), but the percentage of fertilized eggs that hatched (*r_s_* = −0.246, *N* = 101, *P*<0.013; Fig. 2B), the number of tadpoles (*r_s_* = −0.378, *N* = 101, *P*<0.001; Fig. 2C), and the percentage of tadpoles that metamorphosed into toadlets (*r_s_* = −0.499, *N* = 101, *P*<0.001; Fig. 2D) all decreased with increasing divergence between species in hybrid crosses.

**Figure 3:**
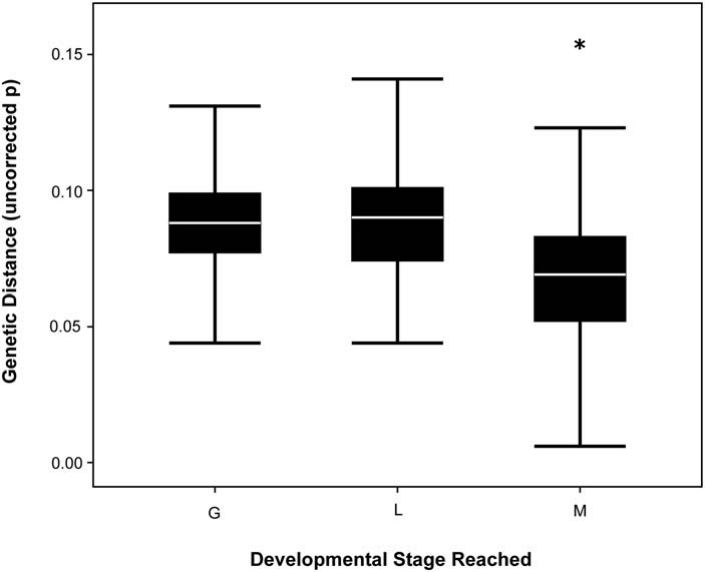
Boxplots of genetic distance (uncorrected p) compared to the latest developmental stage reached in hybrid crosses. Upper and lower whiskers represent 75^th^ and 25^th^ quartiles and white line represents the median genetic distance. G = gastrula; L = larvae/tadpoles; and M = metamorphosis into froglets. Sample sizes are shown above each category of developmental stage. * represents results of multiple comparison tests for each pairwise comparison.

We tested the relationship between developmental stage reached by hybrid offspring and genetic divergence between parental species and found that development of hybrids proceeds further when the parents are more genetically similar (*F*_2,489_ = 62.729; *P*<0.0001; Fig. 3). The genetic distance for crosses that reached gastrula or larval stages was the same (average distance for gastrula stage = 0.089±.001, *N* = 257; average distance for larval stage = 0.089±.002, *N* = 145), but the average distance for crosses that reached metamorphosis, decreased 0.003 substitutions/site (average distance = 0.062±.003, *N* = 90; Bonferroni corrected *P* for multiple comparisons, *P*<0.0001 for all comparisons). Collectively, these results illustrate that crosses involving more similar species reach later stages of development compared to crosses between more divergent species.

The vast majority of crosses (92.1%) show some degree of postzygotic reproductive isolation (IPO>0.00), and the average genetic distance at which a given level of postzygotic isolation was reached can be seen in Table 2. The average genetic distance for levels of IPO between 0.0 and 0.50 were similar, but there was a significant increase in genetic distance between IPO 0.60 and IPO 0.80 (Table 2). Interestingly, genetic distance between species pairs with no postzygotic isolation (IPO = 0) ranged from 0.004–0.123, while species pairs with full postzygotic isolation (IPO = 1) ranged from 0.027–0.152, suggesting that postzygotic isolation can be both weak between distantly related species pairs, and strong between closely related species pairs.

**Table 2:** Average genetic distance for each reproductive isolation index level.

Reproductive Isolation Index	Mean genetic distance±SE (N)	
0.00	0.045±0.035 (2)	A
0.20	0.043±0.007 (3)	A
0.40	0.043±0.003 (9)	A
0.50	0.046±0.007 (7)	A
0.60	0.058±0.002 (11)	A
0.80	0.073±0.002 (24)	B
1.00	0.076±0.003 (45)	B
Mean	0.067±0.002 (101)	

Levels grouped by letters are not significantly different as determined by Scheffe's F test.

### Sex Ratios

Blair preserved many samples of hybrid offspring from the experimental crosses and these specimens are housed in the Texas Natural History Collection at the University of Texas. We visually examined the sex of all preserved hybrid specimens (*N* = 869), and used these data to make inferences about biased sex ratios in hybrid offspring. Biased sex ratios are an important component of reproductive isolation research as manifested by Haldane's rule, in which the heterogametic sex is either absent, rare, or sterile, and remains one of the few rules of speciation [25]. Hybrid specimens were dissected and the gonads were examined to determine the sex of each individual and to assess the frequency of malformed reproductive organs in hybrids. We evaluated each cross for statistical significance, and scored them as cases of complete inviability (one sex is entirely absent) or quantitative inviability (a statistically significant bias towards one sex).

We observed significantly more males than females (males: *N* = 501 females: *N* = 368; *G* = 20.44, df = 1, *P*<0.001) among all preserved hybrid specimens. Among the statistically confirmed cases of inviability, females were the affected sex in 71% (5/7) of crosses, while males were the affected sex in 29% (2/7) of crosses. Considering all crosses (including those for which there was evidence of asymmetry, but insufficient numbers of offspring to provide a statistical test of sex ratios), females were the affected sex in 70% (65/93) of crosses and males were the affected sex in 30% (28/93) of crosses (Table 3).

**Table 3:** Cases of Haldane's Rule for inviability in toads.

	Unconfirmed cases	Quantitative cases	Complete cases	Total
Females affected	60	1	4	65
Males affected	26	1	1	28
Neither affected	70	–	–	70

“Unconfirmed cases” are those crosses in which one sex is present while the other is absent, but sample sizes are too low to evaluate statistically. “Quantitative cases” are crosses that showed a statistical bias in the number of individuals for a particular sex. “Complete cases” are those crosses that resulted in zero offspring of one sex and more than six offspring of the opposite sex. The total number of crosses used to analyze the operation of Haldane's rule was 93 (65 cases in which females are the affected sex and 28 in which males are the affected sex).

**Figure 4:**
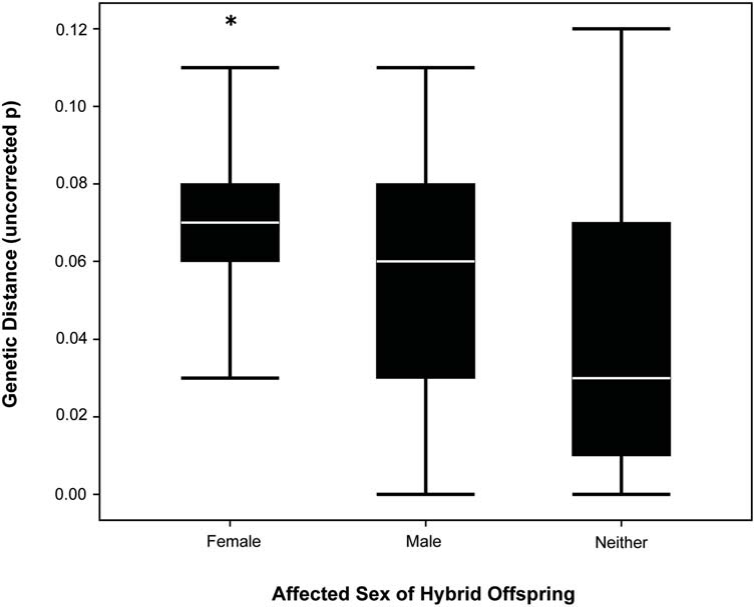
Boxplots of genetic distance compared to negatively affected sex in hybrid crossings. Upper and lower whiskers represent 75^th^ and 25^th^ quartiles respectively. White bars represent median genetic distance for each group. * represents results of multiple comparison tests for each pairwise comparison.

Crosses resulting in inviable hybrid females had higher genetic divergence than crosses that resulted in inviable males (Bonferroni corrected *P* for multiple comparisons, Mann-Whitney *U* = 1362.5, *z* = −2.374, 2-tailed *P*<.018; Fig. 4) and crosses that resulted in viable offspring of both sexes (Bonferroni corrected *P* for multiple comparisons, Mann-Whitney *U* = 1564, *z* = −4.369, 2-tailed *P*<.001; Fig. 4). The average genetic distance for crosses resulting in inviable males was not significantly different from crosses resulting in offspring of both sexes (Bonferroni corrected *P* for multiple comparisons, Mann-Whitney *U* = 1082.5, *z* = −1.769, 2-tailed *P*<0.07; Fig. 4). It should be noted that crosses resulting in offspring of both sexes cover a wide range of genetic distances, as do crosses resulting in only male or female offspring (neither sex affected; average uncorrected p = 0.041±0.004, range = 0.00–0.12; males affected; average uncorrected p = 0.053±0.005, range = 0.00–0.11; females affected; average uncorrected p = 0.065±0.003, range = 0.00–0.12; Fig. 4).

### Patterns of Sterility

Blair determined hybrid fertility by backcrossing hybrids with parental species. If no development occurred, these hybrids were deemed sterile and if offspring developed, hybrids were deemed fertile. These data allow us to examine patterns of sterility for both hybrid males and hybrid females and relate these patterns to divergence between species.

In 38 hybrid male test crosses, 53% (*N* = 20) of males were sterile, and we find no difference in the number of sterile or fertile males (20 sterile vs. 18 fertile; *G* = 0.104; df = 1; *P*>0.05). Despite nearly equivalent numbers of sterile and fertile hybrid males, the average genetic distance for crosses that produced sterile hybrid males (0.064±0.006) was significantly higher than the average genetic distance for crosses that produced fertile hybrid males (0.039±0.005; Mann-Whitney *U* = 77.5, *z* = −2.823, *N* = 38, *P*<0.004), suggesting that genetic divergence predicts hybrid male sterility in toads. After examining 501 male hybrid specimens, we found that 1% (*N* = 5) of males had regressed or malformed testis. Interestingly, 1.6% (*N* = 8) of hybrid males contained eggs in association with the Bidder's organ. The Bidder's organ is a unique organ present in male bufonids composed of rudimentary ovarian tissue that normally does not produce functional eggs [46].

Data were available for 10 crosses involving three types of hybrid females (*Bufo woodhousii×B. hemiophrys*; *B. terrestris×B. hemiophrys*; and *B. terrestris×B. woodhousii*). These hybrid females produced offspring of both sexes and therefore were completely fertile. Interestingly, hybrid males for these same species pairs were also completely fertile. These species are closely related (average uncorrected p = 0.01), and therefore contradict the notion that Haldane's rule operates during the earliest stages of divergence/speciation [1]. Additionally, we found that 1.4% (*N* = 5) of the 369 hybrid females we examined possessed regressed or malformed ovaries.

### Ploidy of Hybrid Offspring

The relationship between abnormal chromosome number in hybrids and genetic divergence has not been examined in animals, and Blair's data offer a unique opportunity to examine this question given the karyotypic analyses of hybrids by Bogart [44]. An analysis of ploidy as related to fertility pattern is also of interest as polyploid animals frequently suffer fertility defects.

We found a positive relationship between the degree of ploidy and genetic distance. Crosses that resulted in triploid and pentaploid karyotypes had an average increase of about 0.02 substitutions/site (diploid average uncorrected p = 0.0713±0.028, *N* = 90; triploid average uncorrected p = 0.0881±0.0150, *N* = 20; pentaploid average uncorrected p = 0.0816±0.0137; *N* = 5; *F*_2,112_ = 3.70; *P* = 0.02). We found no relationship between the level of ploidy for species pairs in a given cross and the likelihood of producing viable hybrid offspring (Fisher's Exact Test, *P* = 0.2250). Regarding sterility, only six crosses that were scored for fertility were also karyotyped. Among these six crosses, one was a sterile triploid, two were sterile diploids, and three were fertile diploids. In summary, higher levels of genetic divergence increase the incidence of polyploidization resulting from hybridization (allopolyploidization), but polyploidization does not appear to result in inviability or sterility.

## Discussion

Investigating patterns of reproductive isolation within diverse taxonomic groups provides powerful evidence for general phenomena related to the characteristics of speciation [47]. Toads, a species-rich group of anurans, are particularly germane to this goal given the apparent discrepancies regarding patterns of reproductive isolation [1], [20], [48]–[50] and the diversity of sex determination systems found in frogs and toads (reviewed in [51]).

Our analyses demonstrate several features consistent with the emerging trends of reproductive isolation. First, intrinsic postzygotic reproductive isolation shows a positive correlation with genetic divergence. Second, as genetic divergence increases, surrogates of fitness such as the percentage of hatching embryos, the number of tadpole larvae produced, and the percentage of larvae that metamorphose all decrease in hybrid crosses. These results are congruent with a previous analysis of reproductive isolation among seven genera of anurans [15], and with comparative studies in other organisms [5]–[24]. In general, Blair's data support the idea that intrinsic postzygotic reproductive isolation is a gradual and likely outcome between diverging populations.

While some aspects match the broad patterns found in other comparative studies, there are several results that contradict the generalities of reproductive isolation. We found that the percentage of eggs fertilized in hybrid crosses was not correlated with genetic divergence, suggesting that incompatibility between toad species arises after the fertilization process. Previous analyses [15] found that the percentage of eggs fertilized decreased with increasing genetic divergence. Our results suggest that interactions between sperm and egg require substantial divergence to prevent fertilization, and this level of divergence has not yet accumulated between many toad species.

We also found relatively high levels of divergence (average uncorrected p = 0.089) were required for hybrid offspring to die during early developmental stages, and that smaller degrees of divergence allowed more advanced stages of development (Fig. 3). Early development in amphibians and other organisms is regulated by maternally loaded RNA transcripts until the point of zygotic activation during the maternal-zygotic transition. The maternal-zygotic transition generally occurs during gastrulation in amphibians, and our data suggest that high levels of divergence are required for crosses to fail during the gastrula stage [52]–[54].

Another unique feature of the toad data was the amount of asymmetry in reciprocal crosses (crosses with an IPO of 0.6). Among interspecific crosses that were performed in both directions, 8% (19/233) produced offspring in only one direction, indicating species-level parent of origin effects. The average genetic distance for these crosses was relatively high (uncorrected p = 0.068±0.002), but this level of divergence does not explain how viable offspring can be produced exclusively in only one direction. Parent of origin effects similar to those in this dataset have also been observed in natural populations of toads and other anuran species [55], [56]. Recently, Bolnick et al. (2008) [57] demonstrate that asymmetric hybrid viability (Darwin's corollary to Haldane's rule) can be explained by differential rate evolution of mitochondrial genomes between species. However, these types of asymmetries may even be expected under a Dobzhansky-Muller model of incompatibility accumulation [58]. This suggests that mechanisms like mitochondrial rate evolution or the accumulation of incompatibilities could promote parent of origin effects that may influence reproductive isolation in anurans [59], [60].

### Implications for Haldane's Rule

Haldane's rule, where the heterogametic sex (XY or ZW) suffers the most dysfunctional effects of hybridization, is one of the broadest generalizations about the process of speciation. Examining biases in sex ratios is therefore important for understanding the implications of Haldane's rule in toads and anurans in general.

One particularly interesting aspect about frogs and toads is that sex determination systems are known to switch among different lineages [61]. While the implications of labile sex determination for Haldane's rule are unclear, one group of frogs (*Xenopus*) contradicts Haldane's rule because hybrid males are sterile, even though females are the heterogametic sex [1], [48], [49], [62]–[64].

Toads possess homomorphic sex chromosomes and karyotype analyses cannot provide information about the sex determination mechanism for toad species. However, sex-reversal experiments have confirmed a ZW sex determination system for the European toad (*Bufo bufo*), thus establishing females as the heterogametic sex for at least one toad species [51], [65], [66]. If we assume that all bufonids possess a ZW sex determination system, then asymmetrical inviability or sterility of hybrid offspring should affect females according to Haldane's rule. Our results confirm Haldane's rule for inviability in that females are underrepresented among the total number of preserved hybrid specimens and hybrid females were the affected sex in about 70% of hybrid crosses showing asymmetrical inviability. Additionally, interspecific divergence was higher in crosses in which females were inviable. While the genetic divergence and female inviability data showed some concordance with the predictions of Haldane's rule, males were the affected sex in 30% of crosses. These data suggest that interesting mechanisms may be operating in toads because the number of exceptions to Haldane's rule for inviability is far higher compared to other organisms. For example, in butterflies and moths, only 4% (3/84) of all species pairs are exceptions to Haldane's rule for inviability [14] compared to the 30% observed in toads.

Another interesting observation is that nearly half (43%) of hybrid crosses produced offspring of both sexes despite extensive divergence between parental species (average uncorrected *p* = 0.051±0.004, ranging from 0.00 to 0.12). Hybrid crosses that produced offspring of both sexes should have low genetic divergences over a relatively narrow range [1], but this is not the case in *Bufo*. In fact, the range of genetic distance among crosses that produced only males, only females, or offspring of both sexes was nearly identical (Fig. 4).

Toads are also unusual with regard to patterns of sterility. Within the subset of hybrids used in further test crosses, 53% of hybrid male toads were sterile and no hybrid females were sterile, contradicting the operation of Haldane's rule for sterility (assuming a ZW sex determination system). Additionally, genetic divergence was a predictor of hybrid male sterility with greater divergence between parental species producing sterile hybrid males and less divergence producing fertile hybrid males. While a small number of exceptions to Haldane's rule have been documented in other organisms [59], the sterility patterns seen in toads are exceptional. For example, 97% (29/30) of butterflies and moths and 98% (112/114) of fruit flies (*Drosophila*) obey Haldane's rule for sterility [1], [14].

Finally, hybridization between three closely related species (*B. hemiophrys*, *B. terrestris*, and *B. woodhousii*) provides further evidence for unusual patterns regarding Haldane's rule in toads. Both hybrid sexes from these crosses were completely fertile, suggesting that Haldane's rule is not operating in toads at the earliest stages of divergence [27]. These results, along with the seemingly high number of exceptions to Haldane's rule for inviability, suggest several hypotheses to explain postzygotic isolation mechanisms in toads.

### Polyploidy and Sterility

Levels of ploidy in hybrid toads could account for sterility in hybrid males when they should be fertile. For example, polyploidy resulting from interspecific hybridization could produce abnormal phenotypic effects in hybrids, and therefore we might predict a relationship between ploidy and degree of sterility or inviability. Genetic divergence was positively correlated with degree of ploidy, with greater levels of divergence associated with higher levels of ploidy, but interestingly, the probability of producing inviable or sterile hybrid offspring was not correlated with ploidy level. These data suggest that ploidy is not a key predictor of sterility even though there is an association between genetic divergence and ploidy. Furthermore, Bogart [44] mentioned at least one instance of triploid hybrid males that were completely fertile, suggesting again that ploidy cannot fully explain sterility patterns in toads. To summarize, we conclude that these data suggest a complex relationship between ploidy and hybrid abnormalities that, while coarsely related to genetic divergence, does not explain the general patterns regarding fertility in toads. The mechanisms for this relationship should be an excellent subject for future work.

### Additional Hypotheses

It is clear that the inviability and sterility patterns observed in toads raise interesting questions about the operation of Haldane's rule. As mentioned above, anurans are remarkable in that genetic sex determination is labile across clades [51], [61] and this might explain at least some of the unusual trends. For example, among anurans there are species with XX/XY, ZZ/ZW, and OO/OW sex determination, including one well-studied example in which XY and ZW sex determination systems occur among different populations of a single species (*Rana rugosa*; [67]–[69]). While there is currently no evidence for multiple sex determination systems in *Bufo*, the lability of genetic sex determination in amphibians suggests that this could be a viable explanation for why our results differ from the expectations of Haldane's rule. Table 3 shows that Haldane's rule operates correctly under the assumptions of a ZW sex determination system in 70% of crosses with asymmetrical inviability (i.e. females are inviable and males are viable), and it operates correctly under the assumption of an XY sex determination system in 30% of crosses with asymmetrical inviability (i.e. males are inviable and females are viable). If both ZW and XY sex determination systems do occur within the family Bufonidae, an important point to consider is that our data should contain crosses between ZW and XY species. While relatively little is known about the consequences of hybrid matings between species with different sex determination mechanisms, it has been shown that *Rana rugosa* (in which both ZW and XY sex determination occur within the same species) can produce viable WY genotypes in crosses between XY and ZW individuals [68], [70]. Offspring with the WY genotype show a heavily female-biased (55 of 57) sex ratio [70]. If species crosses within our data are between individuals possessing differing sex determination systems, it is reasonable to expect WY genotype females in the data, and this would be consistent with the elevated numbers of crosses in which males were the affected sex. However, the ultimate test of this hypothesis requires establishing the sex determination system for additional species and correlating these data with the hybrid cross dataset.

An alternative hypothesis is that the exceptions to Haldane's rule that we observe in toads reflect specific mechanisms that generate the rule itself. Much evidence suggests that Haldane's rule can be explained primarily by two mechanisms: dominance effects or faster-male evolution (reviewed in [1]). The dominance hypothesis states that alleles causing hybrid dysfunction are partially recessive, and the heterogametic sex (XY or ZW) is fully exposed to the deleterious effects of these recessive alleles [71]. Dominance could be the most likely explanation producing Haldane's rule for inviability in toads as the majority of crosses result in detrimental effects on females. The faster-male evolution hypothesis suggests that strong sexual selection on male expressed genes increases divergence at these loci, and/or components related to the process of spermatogenesis are sensitive to a hybrid genetic background, resulting in hybrid male sterility [72], [73]. Dominance effects alone cannot explain hybrid male sterility in a ZW sex determination system because females, not males, should be exposed to deleterious recessive alleles. However, faster-male evolution can explain hybrid male sterility, as this hypothesis does not depend on sex determination system.

Faster-male evolution should produce dysfunctional males, whether males or females are the heterogametic sex in hybrid crosses. Heterogametic females should be expected to closely adhere to Haldane's rule because they are exposed to both cytoplasmic/maternal effect incompatibilities and sex chromosome-autosome incompatibilities [60]. Interestingly, several crosses in our dataset result in viable female hybrid offspring and sterile male offspring. This pattern can be explained under the assumption of faster-male evolution, as exceptions to Haldane's rule in organisms with ZW sex determination are expected to occur when faster-male evolution is operating [73]. Evidence suggests that faster-male evolution is more likely to contribute to hybrid sterility than to inviability [12], [74]; however, our data contain apparent exceptions to Haldane's rule involving both sterility and inviability. These patterns could be due to asymmetric cytoplasmic/maternal effects leading to unusually low occurrences of hybrid female inviability, and faster-male evolution effects leading to increased levels of hybrid male sterility. Our results suggest that faster-male evolution could explain cases of hybrid male sterility in toads as seen with other anurans [48]–[50], [75].

One mechanism that may result in faster-male evolution within the genus *Bufo*, and frogs in general, is sexual selection on male advertisement vocalizations. Male toads, like most anurans, produce an advertisement vocalization used to attract conspecific females to breeding sites for mating. Male advertisement vocalizations therefore act as premating isolating mechanisms, and often exhibit character displacement in areas of species overlap [76]–[81].

One of the most well documented examples of divergent advertisement call displacement occurs among the members of the *Bufo americanus* group. Several authors have found that members of this group often occur in sympatry, and advertisement calls for each species are subtly different, a possible prezygotic isolating mechanism [33]–[38], [76], [77], [81]–[83]. There is little to no postzygotic reproductive isolation among any of the members of this group, and Blair [40] used hybrid offspring from crosses within this group in several further test crosses resulting in viable and, in many cases, fertile F_2_ offspring from hybrid crosses and parental backcrosses. This lack of reproductive isolation, coupled with sympatric distributions, should result in natural hybridization among the members of this group; however, lineages appear to remain relatively distinct. Strong prezygotic isolating mechanisms, such as male advertisement call, may provide an explanation for this phenomenon. Although we cannot extract evidence of the strength of prezygotic isolation between toad species from the current dataset, abundant evidence from anuran vocalization studies suggests that prezygotic isolation could be quite important, and may have driven speciation in this group in a manner similar to that of birds [16], [76]–[78], [80]. Why birds mostly follow Haldane's rule, whereas toads contradict several predictions of Haldane's rule raises an interesting question. While studies of Haldane's rule have included a small, but diverse phylogenetic representation of organisms (flies, birds, butterflies and moths, and mammals), perhaps such a limited taxonomic sample has lead to generalizations for a pattern that some organisms ignore due to other mechanisms (e.g. sex determination lability). Toads as well as another group of frogs (*Xenopus*) both exhibit unusual patterns with regard to Haldane's rule [49].

### Summary

Our analyses of reproductive isolation among toads raise important questions about the broad features of speciation due to the fact that toads deviate from these patterns in several ways. First, we do not see a relationship between fertilization rate and genetic divergence, suggesting that substantial genetic divergence is required in order to achieve postzygotic reproductive isolation between toad species. Second, the patterns we find with regard to sex ratios deviate from previous analyses. Hybrid females are fertile even in the earliest stages of speciation, there is relatively little differential inviability or sterility between males and females despite substantial genetic divergence, and there is no relationship between ploidy and degree of sterility in males. Definitive conclusions regarding Haldane's rule await confirmation of the sex-determination system in additional species of toads. However, given results from other analyses of Haldane's rule in anurans, more surprises are likely to be revealed in investigations of reproductive isolation in frogs and toads.

## Materials and Methods

We compiled the data found in Appendix H of Blair (1972) [40], consisting of 1,934 crosses between 92 species of *Bufo*. Crosses were generally made by *in vitro* fertilization using squashes of testis from one species to fertilize the eggs of another species in a petri dish. After fertilization, eggs in petri dishes were flooded with aged or pond water and placed in enamel pans (30.4×45.7 cm) containing aged or pond water. A subset of eggs was examined for cleavage. After hatching, the tadpoles were spread out (approximately 20 individuals per pan) to avoid crowding. Eggs were housed in small containers, and tadpoles were raised through metamorphosis to adulthood. Various measures of reproductive isolation were quantified based on these *in vitro* fertilizations including; 1) the percentage of fertilized eggs; 2) percentage of hatching embryos; 3) number of tadpoles produced; 4) percentage metamorphosed; 5) fertility in backcross analysis; and 6) the stage at which eggs ceased to develop. Many interspecific crosses were represented by multiple replicates, and in these cases, we calculated the mean for each measure of reproductive isolation and used these mean values in subsequent analyses.

### Calculation of Postzygotic Isolation Indices

Indices of postzygotic isolation were calculated following the method of Zouros (1973) [83], Coyne and Orr (1989) [9], Sasa et al. (1998) [15], and Presgraves (2002) [14]. For reciprocal crosses, we counted the number of sexes that were completely inviable or sterile, giving a value ranging from 0 (both sexes viable/fertile in reciprocal crosses) to 4 (both sexes inviable/sterile in reciprocal crosses). Dividing this number by 4 produces an index of postzygotic isolation (IPO) ranging from 0 (no isolation between species) to 1 (complete isolation between species). Unidirectional crosses were calculated in a similar manner with the exception of dividing by 2 instead of 4, again resulting in an IPO value ranging from 0 to 1 [15].

Examination of the data frequently showed one direction of the cross produced offspring, while the reciprocal cross for the same species pair failed to produce offspring. To account for this pattern, we modified the above-mentioned method to obtain postzygotic isolation indices for subsequent analyses. For crosses that were performed in both directions, we added an additional category to the traditional postzygotic isolation index [9], [10], [84] in order to account for species-level parent of origin effects operating in toads (Table 1).

### Genetic Distance

Sequence data for the mitochondrial DNA fragment 12S–16S were downloaded from Genbank for 69 of the 92 species included in Blair's original dataset (Appendix S1; [29], [42], [43]). Alignments were performed manually using the program Se-Al version 2.0a11 [85], and corrected for secondary structure using models obtained from Pauly et al. [41]. Genetic distance was calculated for all pairwise comparisons using the uncorrected p and the Kimura 2-parameter functions in PAUP* 4.0 Beta [86]. The Kimura 2-parameter distance estimate was used to correct for multiple substitutions while considering transition and transversion substitution rates [87]. The use of either uncorrected p or Kimura 2-parameter did not affect the results, so we present only the uncorrected p divergence estimates. After averaging data from replicate crosses, and excluding crosses for which IPO value and/or genetic distance estimates were unavailable, the final dataset consisted of 680 crosses.

### Phylogenetic Correction

To ensure that our observations of reproductive isolation were phylogenetically and statistically independent, we employed Coyne and Orr's [9], [10] modification of Felsenstein's independent contrasts method [88]. First, we generated a phylogeny for our full molecular dataset (69 species) using maximum parsimony methods in order to examine the phylogenetic relationships among species in our dataset. We then averaged the genetic distances and other measures of postzygotic isolation for species pairs that were not independent to produce a single, independent comparison. Implementation of this modification reduced the dataset from 680 nonindependent crosses to 101 independent crosses.

Additionally, we examined the phylogenetic tree we generated for concordance with previously published phylogenies [30], [42], [43] in order to look for any disagreements or unusual relationships that may be indicative of introgression of mitochondrial DNA (mtDNA) due to natural hybridization. The phylogeny (based on the same mtDNA fragment from multiple studies and, in some cases, multiple individuals of a given species) largely agreed with all recent bufonid phylogenies. No unusual relationships or indirect evidence of hybridization were found, suggesting that the mtDNA data is not affected by natural hybridization.

### Haldane's Rule

Blair preserved many samples of hybrid offspring from the experimental crosses and these specimens are currently housed in the Texas Natural History Collection at the University of Texas. We visually examined the sex of all preserved hybrid specimens (*N* = 869), and used these data to make inferences about the operation of Haldane's rule in the family Bufonidae. Hybrid specimens were dissected, and the gonads were examined to determine the sex of each individual and to assess the frequency of malformed reproductive organs in hybrids. While these certainly do not represent all of the hybrid offspring from Blair's experiments, they do represent a wide spectrum of interspecific crosses, and the majority produced more than one adult hybrid offspring. We confirmed and counted the number of males and females from each cross, combined data from replicate crosses, and used these data to examine the operation of Haldane's rule for inviability in the family Bufonidae.

Following Presgraves (2002) [14], we evaluated each cross for statistical significance, and scored them as cases of complete inviability (one sex is entirely absent) or quantitative inviability (a statistically significant bias towards one sex). Due to relatively low sample sizes for the numbers of each sex resulting from a given cross (ranging from *N* = 0 to 18), we used a binomial distribution test to evaluate statistical significance [89]. This allowed us to determine threshold values for significant differences in the numbers of males and females produced from each cross. For a complete case of inviability to be considered statistically significant, one sex had to have at least six surviving adults, while the other sex had zero survivors. Quantitative cases (cases where both sexes were present, but there was a significant bias in the numbers of one sex) were also analyzed for statistical significance using a binomial distribution test.

Many crosses had sample sizes too low to be evaluated statistically using the aforementioned method. We tallied the crosses that did not meet the statistical criteria for sample size, and placed them in three distinct categories: only female offspring present, only male offspring present, and both sexes present.

Inferences regarding Haldane's rule for sterility were produced by examining the results of F1 hybrid test crosses. This dataset was smaller compared to the inviability dataset but still provides estimates for the operation of Haldane's rule for sterility in toads.

### Ploidy Analyses

Bogart (1972) [44] investigated chromosome content in 50 species and 175 hybrid combinations of *Bufo*, and reported these results in Appendix G of Blair (1972) [40]. Hybrid offspring from Blair's crossing experiments could be placed into one of three categories: diploids, triploids, and pentaploids. We used this data to examine the relationship between ploidy of hybrid offspring and genetic divergence between parental species. We also examined the relationship between ploidy and asymmetrical inviability and sterility of hybrid offspring.

### Statistical Analyses

For the phylogenetically corrected dataset, genetic distance, percentage of eggs fertilized, percentage of embryos hatched, and percentage of metamorphosed larvae were arcsine square-root transformed to adhere to normality assumptions. IPO values were not normally distributed, therefore we used non-parametric Spearman rank correlations for analyses including these values. We used two-tailed, independent samples *t*-tests (corrected for multiple comparisons using Scheffe's F test), and Mann-Whitney *U* tests (corrected for multiple comparisons using Bonferroni correction) in order to evaluate differences between means. All analyses were performed using the program SPSS (SPSS version 11.0.4, SPSS Inc.).

## Supporting Information

Appendix S1 Raw data used in analyses
(0.15 MB XLS)
